# Toxins and Secretion Systems of *Photorhabdus luminescens*

**DOI:** 10.3390/toxins2061250

**Published:** 2010-06-01

**Authors:** Athina Rodou, Dennis O. Ankrah, Christos Stathopoulos

**Affiliations:** Department of Biological Sciences, California State Polytechnic University, Pomona, CA 91768, USA; Email: arodou@csupomona.edu (A.R.); doankrah@csupomona.edu (D.O.A.)

**Keywords:** *Photorhabdus luminescens*, bacterial protein toxins, secretion systems, gram-negative bacteria

## Abstract

*Photorhabdus luminescens* is a nematode-symbiotic, gram negative, bioluminescent bacterium, belonging to the family of *Enterobacteriaceae*. Recent studies show the importance of this bacterium as an alternative source of insecticides, as well as an emerging human pathogen. Various toxins have been identified and characterized in this bacterium. These toxins are classified into four major groups: the toxin complexes (Tcs), the *Photorhabdus* insect related (Pir) proteins, the “makes caterpillars floppy” (Mcf) toxins and the *Photorhabdus* virulence cassettes (PVC); the mechanisms however of toxin secretion are not fully elucidated. Using bioinformatics analysis and comparison against the components of known secretion systems, multiple copies of components of all known secretion systems, except the ones composing a type IV secretion system, were identified throughout the entire genome of the bacterium. This indicates that *Photorhabdus luminescens* has all the necessary means for the secretion of virulence factors, thus it is capable of establishing a microbial infection.

## 1. Introduction

*Photorhabdus luminescens* is a nematode-symbiotic, gram negative, bioluminescent bacterium, belonging to the family of *Enterobacteriaceae*. It is part of the *Photorhadbus* genus, known to have three bacterial species: *Photorhabdus luminescens*, *Photorhabdus temperata* and *Photorhabdus asymbiotica* [1]. The first two species are nematode-symbiotic, while the third one has been isolated from human wounds, suggesting a role of this bacterium as an emerging human pathogen.

*P. luminescens* has a complex life cycle. It is symbiotic to entomopathogenic nematodes of the family *Heterorhabditidiae* [1,2], colonizing in the intestine. *Heterorhabditis* attacks insects at the stage of larvae. Once the nematode, in its infective juvenile stage, enters the bloodstream of the target insect, the bacteria are released and secrete toxins that rapidly kill the insect. Enzymes are also produced from the bacteria that lead to the decomposition of the carcass, providing both the bacteria and the nematodes with enough food for survival and reproduction. Then, new infective juveniles colonized with the bacterium grow, before emerging from the carcass into the soil. Bactericidal products of *P. luminescens* prevent infections of the carcass by other bacteria. 

In order to combine symbiosis with the nematode and pathogenicity with the larva of the insect, *P. luminescens* must produce factors that can do both. The pathogenicity of the bacterium is the result of the presence of pathogenicity islands on its chromosome. In these islands there is a large number of genes that encode for toxins, enzymes, bacteriocins and antibiotics. Secretion of these proteins may happen with the use of different secretion systems, such as the type III secretion system. Genomic islands also encode for genes that are responsible for symbiosis and nematode growth. 

Another characteristic of *P. luminescens* is that it undergoes phase variation [1,3]. There are two phenotypic variants of the bacterium. Phase I or primary variant is found at the infective juvenile stage of the nematode life cycle. At this point, the bacterium produces dyes, antibiotics, lipases, proteases and bioluminescence (due to the *lux* operon). Phase II or secondary variants are the result of *in vitro* cultures, after extended incubation, probably as a response to environmental stress. These variants lack or have reduced levels of the properties of the phase I variants. However, both of the variants show similarities to their pathogenicity.

*P. luminescens* produces a number of toxins. *Tc* toxins [4] are a new class of insecticidal toxins that have been shown to demonstrate both oral and injectable activity with results against the Colorado potato beetle, *Leptinotarsa decemlineata* and *Bemisia tabaci* [5], while other toxins like *Mcf* promote apoptosis in a variety of cells including mammalian ones. This states the potential use of the *P. luminescens* toxins as a replacement of the *Bt* toxins (*Bacillus thuringiensis* toxins) in the agriculture of transgenic crops infected by insects resistant to *Bt* toxins [6]. On the other hand, the fact that human infections with *Photorhabdus* species have been stated before in the USA and Australia [7,8], and the similarity of the *tc* genes of *P. luminescens* with its corresponding genes from *Yersinia pestis* [9], the causative agent of plague, can raise some serious questions on the possibility of a new human pathogen emerging.

## 2. Toxins of *Photorhabdus luminescens*

In order to be able to infect its host and survive, *P. luminescens* must be capable of producing a wide range of proteins, including toxins. The complete genomic analysis of this organism has revealed that it indeed possesses a lot of genes encoding for toxins, proteases and lipases [10]. Four pathogenicity islands were also identified in the *P. luminescens* genome [11,12]; three of them contain genes that encode for toxins, while the last one encodes for a type III secretion system.

In *P. luminescens*, toxins are classified into four major groups: the toxin complexes (Tcs) with a high similarity to the ones found in *Y. pestis* genome [9], the *Photorhabdus* insect related (Pir) proteins, the “makes caterpillars floppy” (Mcf) toxins and the *Photorhabdus* virulence cassettes (PVC) [6,13].

### 2.1. Toxin Complexes (Tcs)

The toxin complexes (Tcs) are encoded by the PAI I (pathogenicity island I) [11,12] and have been identified as high molecular weight insecticidal toxins comprised of multiple subunits [4,6,14]. There are four such complexes, namely Tca, Tcb, Tcc and Tcd, found in different loci. However, *tca* and *tcc* loci encode for several open reading frames (ORFs) [14,15,16], thus producing multiple components per locus; *tcb* and *tcd* are comprised of a single long ORF. Tca, Tcb and Tcc show no overall similarities with other sequences in GenBank; however TccA (one of the three different ORFs of the *tcc* locus) shows similarity to a *Salmonella* protein (SpvA), while TcaC shows similarity to SpvB [16,17]. 

The three complexes show significant similarity to one another; therefore three basic types of genetic elements have been identified: the *tcdA*-like element, equivalent to the combination of *tcaA* and *tcaB*, the *tcdB*-like element, equivalent to the *tcaC* and the *tccC*-like element. *tcdA*-like elements are responsible for establishing primary toxicity, while the *tcdB*/*tccC*-like elements are potentially toxic [18]. Genomic analysis of *P. luminescens W14* has also revealed the presence of cytolytic RTX-like toxins, similar to those secreted by the two-partner system of *Vibrio cholerae* (RtxA-RtxB), *Serratia marcescens* (ShA-ShB), *Erwinia tarda* (EthA-EthB) and *Erwinia chrysanthemi* (HecA-HecB), as well as the presence of novel *tc* loci, based on homology and BLAST search [19]. *tcb* and *tcd* loci encode for proteins of similar sizes as the A and B toxins of *Clostridium difficile*, suggesting similar modes of action between the proteins of the two loci and the ones of the *Clostridium* [17]. *tca* and *tcd* encode for orally active toxins, responsible for the majority of the insecticidal activity in *Manduca secta* [20]. 

The *tc* genes are encoded alongside other genes with putative virulence functions [13,21]. *Tc*-like genes have been identified in both other insect-associated bacteria such as *Serratia entomophila* and non-insect-associated bacteria like some *Pseudomonas* species [4,6]. The insecticidal activity of these complexes is of proteinaceous nature and toxicity is achieved through oral administration [15,17].

### 2.2. Photorhabdus Insect Related (Pir) Toxins

The *Photorhabdus* insect related (Pir) toxins act as binary proteins. They are encoded by the *PirAB* genes, located at *plu4093-4092* (*pirA*) and *plu4437-4436* (*pirB*) loci of *P. luminescens TT01* genome; both proteins are necessary for injectable but not oral activity [6,13]. These proteins show similarities to the δ-endotoxins of *Bacillus thuringiensis*, thus making them a putative substitute for Bt (*Bacillus thuringiensis* toxin) recombinant crops [6]. PirA shows little similarity to known proteins, but its partner PirB shows high homology with the N-terminal region of the pore-forming domain of the Cry2A insecticidal toxin, suggestive of the existence of a similar motif in these binary proteins. PirB also has similarities with a developmentally regulated protein from the beetle *Leptinotarsa decemlineata*; this beetle protein is presumed to have a juvenile hormone esterase (JHE) activity due to the relation of its expression profile during insect development and the levels of juvenile hormone (JH) produced, thus leading to the assumption that PirB may display the same kind of activity. However, such activity is not demonstrated by PirB [22,23]. 

Within the DNA sequences of the encoding genes, enterobacterial repetitive intergenic consensus (ERIC) sequences have been identified [11,24], suggested to be important for mRNA stability.

### 2.3. “Makes Caterpillars Floppy” Toxins

**Figure 1:**
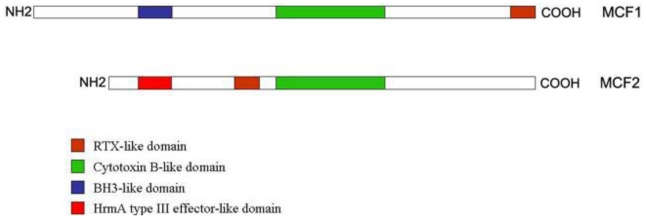
The domain structures of the Mcf proteins. Mcf1 is longer than Mcf2 and in its N-terminus it carries a BH3-like motif, suggestive of its proapoptotic function. In the middle, it has a Cytotoxin B-like motif, highly similar to *Clostridium* cytotoxin B, while in its C-tereminus there is a RTX-like domain. In Mcf2, that RTX-like motif is not found in the C-terminus of the protein. Mcf2 also has a region similar to *Clostridium* cytotoxin B, but it also possesses another domain, similar to HrmA protein (a protein secreted by a type III secretion system) in its N-terminus. Adapted from Dowling *et al.* [26] and Waterfield *et al.* [27].

The “makes caterpillars floppy” toxins 1 (Mcf1) and 2 (Mcf2) act upon injection [6,13,25] and they are encoded by PAI II [11,12], along with other hemagglutinin-like proteins. Mcf1 has been shown to promote apoptosis in the midgut, producing a characteristic “floppy” phenotype in the infected insect, as well as in mammalian cells [26]; it mimics BH3 domain proteins that are found in mitochondria and have proapototic actions [13] as in its N-terminal domain, this protein has a Bcl2-homology 3-like domain (BH3 domain). Its central domain is of hydrophobic character with high similarity of the translocation domain of the *Clostridium difficile* toxin B, while the C-terminal domain of Mcf1 resembles the repeats-in toxin (RTX) like toxins of another bacterium (*Actinobacillus pleuropneumoniae*) (Figure 1) [26]. 

Mcf2 N-terminus shows similarity to a type III secreted protein of a plant pathogen (*Pseudomonas syringae*) but it lacks the BH3-like domain (Figure 1) [27]. *mcf2* locus is located next to a type I secretion system operon. 

Both Mcf1 and Mcf2 have C-terminal domains that support their export via a type I secretion system [23]. These toxins, when expressed recombinantly in *Escherichia coli*, allow the bacterium to survive inside the insect and promote its death and they have been possibly evolved to provide the bacterium with toxicity against different kinds of insects. A different site of action within the insect is also possible for the existence of two different proteins with the same properties [27].

### 2.4. Photorhabdus Virulence Cassettes (PVC)

Given the fact that plenty other toxins should be encoded by the *P. luminescens* genome, in order to make it more efficient in its survival, another group of putative toxins has been identified through homology to antifeeding genes in *Serratia entomophila* [6,13]. These genes in *Serratia* are encoded in a plasmid carrying *tc* homologues and are located in a locus encoding proteins of high similarity to phage proteins [28,29]. 

In *P. luminescens*, multiple copies of those genes have been recognized and are known as “*Photorhabdus* virulence cassettes” (PVCs). These genes show sequence similarities to known toxins such as the Mcf of *P. luminescens* or the toxin A of *C. difficile* [6]. Each of these cassettes encodes for 15-20 proteins, about 30nm wide, that resemble R-type pyocins, a type of bacteriocin. The protein products of the PVCs have no direct antibacterial activity, but do destruct insect hemocytes.. These proteins also show similarities to phage tail and base plate assembly proteins, fimbrial usher and proteins from other pathogenic bacteria [13]. Their loci can be found clustered between a type IV conjugation pilus and the *mukB* locus, a locus involved in plasmid stability. Furthermore, their effector proteins are located always downstream of the PVCs and are flanked by transposon sequences, indicative of a possible mechanism of insertion in the PVC or even their movement among different PVCs [13].

## 3. Secretion Systems and *Photorhabdus luminescens*

Bacteria depend upon their communication with the extracellular environment for survival and interaction with other cells. As such, proteins that are synthesized in the cytoplasm must be transferred to the extracellular space (protein secretion). However, bacterial cells are surrounded by a cell envelope that includes the cell (cytoplasmic) membrane and a cell wall, surrounding it. In Gram negative bacteria, protein secretion involves the crossing of the newly-synthesized protein through two sets of membrane, the inner membrane (IM) and the outer membrane (OM), as well as the space spanning between them, namely the periplasmic space, leading to the development of elaborate secretion mechanisms [30,31].

Secretion can be performed in one or two steps, dividing the secretion pathways in two categories: (i) one-step or Sec-independent pathways, where the protein is secreted through a channel spanning both membranes and the periplasm and (ii) two-step or Sec-dependent pathways, where the protein is initially transferred into the periplasm across the IM with the help of a protein complex embedded in it (Sec translocase), then crosses the periplasm and subsequently is transferred through the OM into the extracellular environment. Up to date, at least seven types of protein secretion pathways have been recognized in Gram-negative bacteria.

### 3.1. One Step Secretion Systems

#### 3.1.1. Type I Secretion System

The type I system is a Sec-independent secretion pathway comprised of three membrane proteins: an ABC (ATP-binding cassette) protein located in the IM that provides the necessary energy amount for secretion and is characterized by conserved regions called Walker A and Walker B motifs, a TolC-like OM channel protein used for the protein export and a membrane fusion protein (MFP) that connects the IM and OM components of the secretion apparatus [32,33].

The genes encoding type I substrates and its components are typically found in the same operon, although there are cases where the components can be found in distant positions on the chromosome. The type I secretion system has been studied extensively in *E. coli* [33,34]. In this case, TolC is recruited by the binding of the substrate (HlyA) to the IM complex. The MFP component (HlyD) joins the cytoplasmic and the periplasmic tunnel that causes an allosteric change. This allows the channel of the TolC to open and release the substrate. Once the transfer is completed, the complex disengages and TolC returns to its closed state.

Bioinformatics analysis in the *P. luminescens laumondii* TT01 genome has revealed that this bacterium possesses multiple copies of this system. More specifically, six putative type I systems (Table 1) were identified. The ABC and MFP proteins are present in all six systems, with three TolC homologues. All ABC and MFP genes are located adjacent to one another and have the nucleotide-binding Walker A and Walker B motifs [35], proving the ATP used by these systems.

#### 3.1.2. Type III Secretion System

Type III secretion system has the unique ability of transporting its effectors directly into the host cell cytosol, by forming a needle-like structure. The apparatus itself is composed of 20–25 [36,37] proteins that are highly conserved. Most of the type III secretion system components are homologous to the ones implicated in the flagellar biosynthesis [37].

The apparatus is comprised of structures on the bacterial surface, an inner membrane ring and an OM ring [36]. Needle-like structures have been identified in *Yersinia* species, which type III secretion system is used as a prototype of that kind of secretion mechanism (*ysc* gene cluster), *Salmonella, Shigella* and *Escherichia* [38,39].

Phylogenetic analysis of the species known to carry type III secretion system genes has revealed seven different families of type III secretion systems, each one of them presenting highly conserved genes and cluster organization. *P. luminescens* type III secretion system has been identified and it is categorized in the same family as the Ysc type III secretion system of *Yersinia* species. In the same family belong the type III systems of *Pseudomonas aeruginosa, Aeromonas* species, *Vibrio parahaemolyticus, Bordetella* species and *Desulfovibrio vulgaris* [39]. Two type III secretion systems were revealed for the *Photorhabdus luminescens*: the Sct type III secretion system and a type III flagellar assembly system system (Supplementary Table 1). A genomic comparison between *P. luminescens* and the emerging human pathogen *P. asymbiotica* shows that the latter possesses a third type III secretion system, which is however located in a second type III secretion system island (in *P. luminescens* genome, there is only one type III secretion system encoding operon, the effector proteins of which facilitate inhibition of phagocytosis of the bacterium by hemocytes) [23]. The acquisition of the second type III secretion system island, which resembles the one of *Vibrio parahaemolyticus*, by P. asymbiotica may contribute to its ability to infect humans [23].

**Table 1:** Type I secretion system components identified in the genome of *P. luminescens*. The percentage of identity was acquired by BLAST search against known system components; in parenthesis, the organism and the corresponding protein with the highest percentage of identity are provided.

Components	NCBI Accession Number	Number of amino acids	Gene locus on *P. luminescens* chromosome	Identity (%)	Function/Structure
ABC 1	NP_927979	711 aa	726,383–728,518	57% (*Bordetella pertussis* CyaB)	Hypothetical protein similar to toxin secretion ATP-binding protein
MFP 1	NP_927980	471 aa	728,660–730,075	36% (*Bordetella pertussis* CyaD)	Hypothetical protein similar to hemolysin export system MFP HlyD of *E. coli*
ABC 2	NP_930357	706 aa	3,664,486–3,666,606	46.4% (*Bordetella pertussis* CyaB)	Hypothetical protein similar to toxin secretion transporter
	NP_930359	719 aa	3,667,994–3,670,153	44.4% (*Bordetella pertussis* CyaB)	Hypothetical protein similar to toxin secretion transporter
MFP 2	NP_93058	462 aa	3,666,606–3,667,994	36% (*Bordetella pertussis* CyaD)	Hypothetical protein similar to toxin secretion transporter (MFP)
ABC 3	NP_928641	716 aa	1,546,913–1,549,063	42.3% (*Escherichia coli* HlyB)	Hypothetical protein similar to unknown protein VC1446 of *V. cholerae*, probable RTX toxin ABC transporter protein
	NP_928643	701 aa	1,550,414–1,552,519	41.8% (*Escherichia coli* HlyB)	RtxB (ABC transporter)
MFP 3	NP_928642	451 aa	1,549,066–1,550,421	32.8% (*Escherichia coli* HlyD)	RTX toxin ABC transporter protein (MFP) RtxD
ABC 4	NP_928002	576 aa	754,786–756,516	70.4% (*Serratia marcescens* LipB)	ATP-binding protein PrtB
MFP 4	NP_928003	444 aa	756,577–757,911	62.7% (*Serratia marcescens* LipC)	MFP PrtC
TolC 1	NP_931154	457 aa	4,641,003–4,642,376	64.7% (*Escherichia coli* TolC)	Outer mebrane channel protein
TolC 2	NP_928004	458 aa	757,911–759,287	50.4% (*Serratia marcescens* LipD)	Outer membrane protein
TolC 3	NP_929881	492 aa	3,099,833–3,101,311	18.9% (*Serratia marcescens* LipD)	Hypothetical protein similar to outer membrane factor of ABC transport system

#### 3.1.3. Type IV Secretion System

Type IV secretion system is used by either Gram-positive or Gram-negative bacteria for the translocation of macromolecules across the cell envelope [40,41]. It shares an ancestral relationship to conjugation machines [40,41,42,43].

There are three different type IV secretion systems according to their effectors and have been identified in many bacteria including *Agrobacterium tumefaciens* (*VirB/D4*) *Bordetella pertussis* (*Ptl*), *Helicobacter pylori* (*Cag*) and *Legionella pneumophila* (*Dot/Icm*) [40,41,43,44].

The best characterized type IV system is the VirB/D4 of *A. tumefaciens*, thus it serves as a prototype for the system [41,44]. In *P. luminescens*, bioinformatics analysis against known type IV components (*A*. *tumefaciens*-*Vir*, *B. pertussis*-*Ptl* and *L. pneumophila*-*Dot/Icm*) failed to recognize any type IV secretion system homologues.

#### 3.1.4. Type VI Secretion System

Type VI secretion system is a newly identified system. The type VI secretion system gene clusters were found to have an IcmF homologue (IcmF-associated homologue protein-IAHP), therefore justifies the early naming of the system as type IVB secretion system (IcmF is part of the type IV secretion system of *Legionella pneumophila*) [45,46].

Type VI secretion systems have been identified in *Edwardsiella tarda, Burkholderia mallei, Pseudomonas aeruginosa* and *Vibrio cholerae* [46,47,48,49]. This mechanism does not require N-terminal signal sequences [48] and it is tightly regulated at both transcriptional and post-translational levels [45,48]. Partially characterized components of the type VI secretion system, apart from the IcmF homologue, include an ATPase and a regulatory FHA domain protein (FHA = forkhead associated domain found in protein kinases and transcription factors) [45,46]. Generally, the type VI system gene clusters encode for 12-25 proteins and the effector proteins belong to either the Hcp protein family or the VgrG protein family [47,48]. Serine and threonine kinases and phosophatases also participate in the system as amino acid phosphorylation seems to play an important role. The genes encoding for these enzymes are usually located close to the gene encoding for the IcmF-like proteins [46].

In *P. luminescens*, multiple copies of putative type VI secretion systems were identified, most of which include an ATPase with the characteristic Walker A and Walker B motifs and an IcmF-like protein (Supplementary Table 1).

### 3.2. Two Step Secretion Systems

#### 3.2.1. IM Translocation

The IM translocation is mediated by the Sec translocase and/or Twin-arginine translocation (Tat) pathway. The Sec translocase comprises of the SecA, an ATPase, the SecYEG complex, which provides the pore-forming channel throughout the OM and the accessory proteins SecDF, YajC and YidC. SecB serves as a molecular chaperone that brings the preprotein into the Sec translocase [50]. 

The Tat pathway mediates the secretion of folded [51] proteins in contrast to the Sec pathway that secretes unfolded proteins. Its substrate precursors have the characteristic sequence motif of two sequential arginine residues in their signal peptide sequence [52]. The Tat translocase of *E. coli* is comprised of TatA, TatB, TatC and TatE. In the same operon, there is an additional gene, *tatD* that encodes a DNase irrelevant to the Tat pathway [51,52,53]. 

In *P. luminescens*, all components of the Sec translocase and the SecB chaperone, as well as all components of the Tat apparatus and two TatD putative DNase genes are identified. The components found present a high identity to the Sec translocase and the Tat system of *E. coli* (Supplementary Table 2).

#### 3.2.2. OM Translocation

##### 3.2.2.1. Type II Secretion System

The type II secretion system is a two-step pathway that utilizes the Sec or Tat machinery for the translocation of the precursor polypeptide across the IM. In the periplasm, the polypeptide becomes the target of the Gsp (general secretory pathway) machinery. The Gsp machinery is normally comprised of 12–16 components, named by the letters A–O and S and organized into a large operon [54,55].

The OM complex is comprised of a secretin, GspD that is thought to act as the pore of the apparatus. Another protein, called GspS, is a lipoprotein that facilitates the insertion of the secretin into the OM and protects it from proteolytic degradation. GspE is a putative ATP binding protein that bares a Walker A motif and a putative Walker B motif. Proteins GspE, GspF, GspL and GspM interact to create an IM platform. GspM has also been proven to be essential for the stabilization of GspL. GspC and GspN are connecting components, while GspA and GspB form a complex that transfers energy from ATP hydrolysis [56].

In *P. luminescens*, several but not all of the components of the type II secretion apparatus were identified, while more than one of the components were identified on the same gene locus on the chromosome of the bacterium (Supplementary Table 2).

##### 3.2.2.2. Type V Secretion System (Autotransporter Secretion System)

The type V or autotransporter secretion mechanism is one of the simplest and most widespread of all the secretion pathways. Autotransporter polypeptides are large virulent proteins, usually over 100 kDa in size that can mediate their own secretion to the extracellular space. Autotransporter proteins can function as proteases, adhesins, toxins and mucinases that aid Gram-negative bacteria in pathogenesis.

Autotransporters are synthesized as large multi-domain precursors comprised of an N-terminal signal sequence, a functional passenger domain and a C-terminal β-domain used for transport across the OM [30]. In order for an autotransporter polypeptide to be secreted it must first cross the membranes of the cell envelope. To accomplish this task, the N-terminal signal sequence inserts into the Sec translocase and transports the protein into the periplasm. Once periplasmically localized, the signal sequence is cleaved off leaving the passenger domain and the β-domain. The β-domain then inserts into the OM, forming a β barrel pore with a central hydrophilic structure. This pore then allows for the final translocation of the passenger domain to the extracellular environment.

Bioinformatics analysis identified only one putative autotransporter protein in the genome of *P. luminescens* (Supplementary Table 2)*.*

##### 3.2.2.3. Two-Partner Secretion (TPS)

The two-partner secretion (TPS) system is common in gram-negative bacteria and is dedicated to the secretion of large virulent proteins, usually greater than 300 kDa. Proteins secreted via this system are separated into two different polypeptides. The secreted protein is labeled as TpsA, whereas the accessory translocator protein is termed TpsB.

During secretion, TpsB forms a beta-barrel like structure in the outer membrane and allows for TpsA to exit the cell through this pore [57]. TpsA and TpsB both contain an N-terminal signal sequence that is recognized by the Sec translocase present in the IM. Once both proteins are transported across the IM, in a Sec-dependent manner, TpsB forms a β barrel structure by inserting its C-terminal domain into the OM. The remaining polypeptide is localized on the periplasmic side of the OM where it interacts with TpsA and mediates its translocation across it [30]. 

Six pairs of putative TpsA-TpsB proteins were identified in the genome of *P. luminescens*, with high identity to the Tps systems of *Serratia marcescens* (Sh1) and *Bordetella pertussis* (Fha) (Supplementary Table 2).

##### 3.2.2.4. Chaperone-Usher Secretion (CU)

The chaperone-usher pathway is responsible for the secretion of proteins forming rod-like organelles that protrude from the bacterial cell surface. These rod-like proteins are composed of various components of subunits. Two proteins commonly utilized by this pathway are P pili and type I pili. These virulent pilus structures aid pathogenic bacteria in formation of biofilms, interactions with hosts and evasion of host defenses [58]. 

In order for pili subunits to be secreted, each individual subunit enters the periplasm via the Sec translocase complex. Once in the periplasm, each subunit interacts with a chaperone to form a stable chaperone-subunit complex. Once the chaperone-subunit complexes are formed, they associate with an usher present in the OM. The usher forms a ring shaped pore that allows for the addition of the pilus subunits in a tip-to-base polymerization. The usher facilitates this polymerization by recognizing chaperone-subunit complexes according to their final positions in the mature pilus structure [57,58]. 

Eight possible chaperone-usher systems were identified in *P. luminescens* genome. Of those eight, three systems had chaperone-ushers with predicted signal peptides necessary for IM translocation (Supplementary Table 2).

## 4. Conclusions

*P. luminescens* is a nematode-symbiotic bacterium with a complex life cycle. As such, it needs to coordinate symbiosis and pathogenicity, thus the production of factors that can assist in both is necessary. Various toxins have been found in the genome of the bacterium, with both oral and injectible activities. These toxins are categorized into four groups; some of them are part of the pathogenicity islands identified in the *P. luminescens* genome.

Tcs are encoded by PAI I and show no similarity to other known proteins; they show high oral activity. The *Photorhabdus* insect related (Pir) toxins are binary proteins that are similar to the δ-endotoxins of *B. thuringiensis.* Mcf1 and Mcf2 are encoded by PAI II and act upon injection; these toxins have similarities to the *C. difficile* toxin, as well as RTX-like toxins. Finally, PVCs show homology to antifeeding genes in *S. entomophila* and encode proteins resembling types of bacteriocins. 

Secretion of these proteins may happen through different secretion systems. Bioinformatics analysis against components of all known secretion systems revealed the existence of multiple copies of various secretion systems components throughout the genome of the bacterium (Table 2 and Table 3).

**Table 2:** Summary of one-step secretion systems in *P. luminescens*.

Secretion System	# of putative systems identified
Type I	6
Type III	2
Type IV	0
Type VI	4

All, but type IV secretion systems are part of the “armory” of *P. luminescens* for survival and pathogenicity. This indicates that the bacterium possesses all the necessary means for the secretion of virulence factors, thus it is capable to establish a microbial infection.

Both type III and IV are used for the direct delivery of effectors in the host cell, using similar structures, thus assisting in processes such as immune system evasion during infection establishment [23]. This is important during the life cycle of the bacterium for its survival. Since both of the aforementioned systems could serve the same purpose, the presence of two type III secretion systems could counteract/justify the absence of a type IV system. On the other hand, type VI secretion systems (formerly known as type IVB) have also been identified; type VI secretion system is yet another system with similar secretion apparatus and could also justify the absence of a type IV secretion system.

Multiple copies of other secretion systems could be attributed to the variety of toxins, virulence factors and other proteins necessary for survival and infestation, secreted from *P. luminescens*. Further analysis however is necessary in order to identify the exact secretion mechanisms of the known toxins, as well as their exact role in establishing infection. Finally, the information presented in review could help in the identification of new secreted toxins through the identified secretion systems.

**Table 3:** Summary of two-step secretion systems in *P. luminescens*.

Secretion System	# of putative systems identified
Type II	1
Type V	1
TPS	6
Chaperone-Usher	8

## Supplementary Files

Supplementary File 1: PDF-Document (PDF, 197 KB)
