# Supplementary material for: Toxins and Secretion Systems of Photorhabdus luminescens
Source: Toxins (Basel). 2010 Jun 1;2(6):1250–64. doi: 10.3390/toxins2061250 (PMC3153242; doi:10.3390/toxins2061250)
Supplement: Supplementary File 1: — PDF-Document (PDF, 197 KB) [file toxins-02-01250-s001.pdf]

SUPPLEMENTARY MATERIAL

Materials and methods

Bioinformatics analysis. For this project, protein sequences of known components of protein secretion systems were retrieved from the NCBI (National Center for Biotechnology Information) database (www.ncbi.nhm.nih.gov) and they were used as queries in BLAST search (www.ncbi.nhm.nih.gov/blast) against the *P. luminescens,* subspecies *laumondii TT01* (reference number NC 005126).

The retrieved sequences used include the type I secretion components of *Escherichia coli* (Hly), *Erwinia chrysanthemi* (Prt), *Bortetella pertussis* (Cya), *Serratia marcescens* (Lip), the type II secretion components of *Vibrio cholerae* (Eps), *Escherichia coli* (Gsp), *Aeromonas hydrophila* (Exe), *Erwinia chrysanthemi* (Out), *Pseudomonas aeruginosa* (Xcp), *Xanthomonas campestris* (Xps), *Klebsiella oxytoca* (Pul) and *Yersinia enterocolitica* (Yts1), the type III secretion components of *Yersinia pestis* (Ysc) and *Salmonella enterica* (Ssa), the type III flagellar system of *Escherichia coli* (Fli), the type IV secretion components of *Agrobacterium tumefaciens* (Vir), *Bordetella pertussis* (Ptl) and *Legionella pneumophila* (Dot/Icm), the type VI secretion components of *Vibrio cholerae* (VAS) and *Edwardsiella tarda* (Evp), Sec, twin-arginine translocation (Tat), signal recognition peptide (SRP) and signal peptidases (SPases) components of *Escherichia coli,* the chaperone/usher of *Escherichia coli* (Pap) TpsA and TpsB of *Serratia marcescens* (Sh1), *Haemophilus influenzae* (HMW1), *Bordetella pertussis* (Fha), *Erwinia chrysanthemi* (Hec), *Proteus mirabilis* (Hpm), *Edwarsiella tarda* (Eth), *Haemophilus ducreyi* (Hhd and Lps1), *Yersinia pestis* (Ytps1) and the autotransporter proteins from *Haemophilus influenza* (Hia and Hap), *Yersinia enterocolitica* (YadA), *Helicobacter pylori* (VacA), avian pathogenic *Escherichia coli* (Tsh), *Shigella flexneri* (IcsA), *Bordetella pertussis* (BrkA) and *Pseudomonas aeruginosa* (EstA).

The hits from the BLAST search were screened based on the following criteria: a) there was a significantly important sequence identity (≥ 20%), b) the hits were part of the same gene cluster or operon and c) conservedresidues and/or motifs specific to the components were identified. Pairwise sequence alignments for the assessment of the identity percentage between the query and the subject sequences was performed using Needleman-Wunch global alignment algorithm (http://www.ebi.ac.uk/ Tools/ emboss/align/index.html), while the signal peptides were predicted using SignalP (http://www.cbs.dtu.dk/services/SignalP/), screening only for Gram negative bacteria and up to 50 amino acids long. Motifs were searched manually.

Supplementary Table 1. One step secretion systems in *P. luminescens*

System	Components	NCBI Accession Number	Number of amino acids	Gene locus on *P. luminescens* chromosome	% Identity	Function/Structure
	YscV	NP_930967	705 aa	4,429,651-4,431,768	82.3% (*Y. pestis* YscV)	SctV (Inner membrane needle base protein)
	YscN	NP_930973	440 aa	4,434,397-4,435,719	85% (*Y. pestis* YscN)	ATPase
	YscP	NP_930975	376 aa	4,436,192-4,437,322	23.6% (*Y. pestis* YscP)	SctP (Needle length regulator)
	YscQ	NP_930976	310 aa	4,437,319-4,438,251	47.3% (*Y. pestis* YscQ)	ATPase associated protein
	YscR	NP_930977	217 aa	4,438,248-4,438,901	82.5% (*Y. pestis* YscR)	Inner membrane needle base protein
Type III	YscS	NP_930978	88 aa	4,438,904-	77.3% (*Y.*	Inner membrane

				4,439,170	*pestis* YscS)	needle base protein
	YscT	NP_930979	262 aa	4,439,167-4,439,955	67.2% (*Y. pestis* YscT)	SctT (Inner membrane needle base protein)
	YscU	NP_930980	351 aa	4,439,952-4,441,007	68.4% (*Y. pestis* YscU)	SctU (Inner membrane needle base protein)
	YscC	NP_930984	614 aa	4,444,107-4,445,951	64.6% (*Y. pestis* YscC)	SctC (Outer membrane secretin)
	YscD	NP_930985	422 aa	4,445,948-4,447,216	26% (*S. enterica* SsaD)	SctD (Needle component)
	YscF	NP_930987	84 aa	4,447,418-4,447,672	63.2% (*Y. pestis* YscF)	SctF (Needle)
	YscJ	NP_930991	245 aa	4,449,008-4,449,745	71.5% (*Y. pestis* YscJ)	SctJ (MS ring protein)
	YscK	NP_930992	208 aa	4,449,745-4,450,371	47.4% (*Y. pestis* YscK)	SctK (ATPase associated protein)
Type III flagellar system	YscU	NP_929162	383 aa	2,256,412-2,257,563	60.7% (*E. coli* FhlB)	Membrane component
	YscV	NP_929163	693 aa	2,257,556-2,259,637	80.4% (*E. coli* FhlA)	Membrane component
	YscS	NP_929204	89 aa	2,304,907-2,305,176	76.4% (*E. coli* FliQ)	Membrane component
	YscR	NP_929205	244 aa	2,305,197-2,305,931	77.8% (*E. coli* FliP)	Membrane component
	YscQ	NP_929207	139 aa	2,306,417-2,306,836	69.5% (*E. coli* FliN)	Flagellar motor switch protein
	YscN	NP_929212	454 aa	2,310,303-2,311,667	79.2% (*E. coli* FliI)	ATPase
	YscL	NP_929213	243 aa	2,311,667-2,312,371	53.3% (*E. coli* FliH)	Flagellar assembly protein
	YscJ	NP_929215	567 aa	2,313,353-2,315,056	60.6% (*E. coli* FliF)	MS ring protein
	EvpH-1	NP_929544	860 aa	2,684,731-2,687,313	49.9% (*E. tarda* EvpH)	Hypothetical protein similar to ClpA-B type chaperone (ATPase)
	EvpO-1	NP_929545	1169 aa	2,687,306-2,690,815	23.7% (*E. tarda* EvpO)	Some similarities with unknown protein and putative macrophage toxin, IcmF-like protein
	EvpN-1	NP_929546	206 aa	2,690,831-2,691,451	26.2% (*E. tarda* EvpN)	Hypothetical protein similar to unknown protein
	VasE-1	NP_929547	465 aa	2,691,451-2,692,848	24.3% (*V. cholerae* VasE)	Hypothetical protein highly similar to unknown protein
	EvpL-1	NP_929548	225 aa	2,692,852-2,693,529	19.3% (*E. tarda* EvpL)	Predicted component of the type VI protein secretion system
	EvpK-1	NP_929549	367 aa	2,693,522-2,694,625	31% (*E. tarda* EvpK)	Predicted component of the type VI protein

						secretion system
	EvpJ-1	NP_929550	100 aa	2,694,639-2,694,941	70.3% (*E. tarda* EvpJ)	Uncharacterized conserved protein similar to unknown protein
	EvpI-1	NP_929552	668 aa	2,695,569-2,697,575	34% (*E. tarda* EvpI)	Uncharacterized conserved protein similar to VgrG protein
	EvpG-1	NP_929553	336 aa	2,697,600-2,698,610	30.9% (*E. tarda* EvpG)	Hypothetical protein similar to unknown protein
	EvpF-1	NP_929554	603 aa	2,698,601-2,700,412	35.2% (*E. tarda* EvpF)	Hypothetical protein similar to unknown protein
	EvpE-1	NP_929555	147 aa	2,700,912-2,700,860	28.1% (*E. tarda* EvpE)	Hypothetical protein similar to unknown protein
	EvpC-1	NP_929556	164 aa	2,700,912-2,701,406	34.9% (*E. tarda* EvpC)	Hypothetical protein similar to unknown protein
	EvpB-1	NP_929557	493 aa	2,701,463-2,702,944	60.6% (*E. tarda* EvpB)	Hypothetical protein similar to unknown protein
	EvpA-1	NP_929558	183 aa	2,702,952-2,703,503	42.1% (*E. tarda* EvpA)	Hypothetical protein similar to unknown protein
	EvpI-2	NP_927712	631 aa	377,581-379,476	28.7% (*E. tarda* EvpI)	Similar to VrgG protein
	VasK-1	NP_927714	1181 aa	382,431-385,976	37.9% (*V. cholerae* VasK)	Similar to putative macrophage toxin, IcmF-related protein, putative transmembrane protein.
	VasJ-1	NP_927715	477 aa	385,973-387,406	30.5% (*V. cholerae* VasJ)	Similar to unknown protein
Type VI	VasI	NP_927716	215 aa	387,412-388,059	26.7% (*V. cholerae* VasI)	Similar to unknown protein
	EvpH-2	NP_927718	881 aa	388,853-391,498	44.5% (*E. tarda* EvpH)	Similar to ClpA-B type chaperone (ATPase)
	VasF-1	NP_927719	256 aa	391,509-392,279	37.4% (*V. cholerae* VasF)	Highly similar to Unknown protein
	VasE-2	NP_927720	450 aa	399,279-393,631	43.6% (*V. cholerae* VasE)	Highly similar to unknown protein
	VasD	NP_927721	188 aa	393,634-394,200	30.9% (*V. cholerae* VasD)	Similar to unknown protein, predicted component of type VI secretion system
	VasC	NP_927722	428 aa	394,200-395,486	30.4% (*V. cholerae* VasC)	Similar to unknown protein, predicted component of type VI secretion system
	VasB-1	NP_927723	326 aa	395,492-396,472	37.4% (*V. cholerae* VasB)	Similar to unknown protein
	VasA-1	NP_927724	615 aa	396,508-	46.4% (*V.*	Highly similar to

				398,355	*cholerae* VasA)	unknown protein
	EvpB-2	NP_927726	492 aa	398,804-400,282	40.6% (*E. tarda* EvpB)	Highly similar to unknown protein
	EvpA-2	NP_927727	165 aa	400,306-406,803	30.3% (*E. tarda* EvpA)	Highly similar to unknown protein
	Hcp1-1	NP_927728	172 aa	401,704-402,222	77.3% (*V. cholerae* Hcp1)	Highly similar to Hcp protein
	VasB-2	NP_930451	362 aa	3,811,002-3,812,090	23.1% (*V. cholerae* VasB)	Similar to unknown protein
	VasA-2	NP_930452	586 aa	3,812,054-3,813,814	30.6% (*V. cholerae* VasA)	Similar to unknown protein
	VasK-2	NP_930458	1123 aa	3,819,697-3,823,068	17.8% (*V. cholerae* VasK)	Similar to putative protein of *Y. pestis,* IcmF homologue
	EvpI-3	NP_930464	841 aa	3,827,457-3,829,982	20% (*E. tarda* EvpI)	Similar to vgrG related protein
	EvpI-4	NP_930473	799 aa	3,840,506-3,842,905	21.8% (*E. tarda* EvpI)	Similar to vgrG related protein
	VasK-3	NP_930474	1123 aa	3,843,641-3,847,012	17.3% (*V. cholerae* VasK)	Similar to putative protein of *Y. pestis,* IcmF homologue
	VgrG2	NP_930480	842 aa	3,852,594-3,855,122	21.5% (*V. cholerae* VgrG2)	Similar to VrgG protein
	Hcp1-2	NP_930481	163 aa	3,855,299-3,855,770	30.8% (*V. cholerae* Hcp1)	Highly similar to Hcp protein
	VasE-3	NP_930486	449 aa	3,860,551-3,861,900	27% (*V. cholerae* VasE)	Highly similar to unknown protein
	EvpB-3	NP_930487	508 aa	3,861,916-3,863,442	41% (*E. tarda* EvpB)	Highly similar to unknown protein
	EvpA-3	NP_930488	165 aa	3,863,474-3,863,971	31.1% (*E. tarda* EvpA)	Highly similar to unknown protein
	VasL	NP_931381	459 aa	4,911,700-4,913,079	20.4% (*V. cholerae* VasL)	Similar to unknown protein
	VasB-3	NP_931384	346 aa	4,914,086-4,915,126	23% (*V. cholerae* VasB)	Similar to unknown protein
	VasA-3	NP_931385	588 aa	4,915,126-4,916,892	30.1% (*V. cholerae* VasA)	Similar to unknown protein
	VasJ-2	NP_931386	532 aa	4,916,971-4,918,569	20.8% (*V. cholerae* VasJ)	Similar to unknown protein
	Hcp1-3	NP_931392	163 aa	4,925,955-4,926,446	32% (*V. cholerae* Hcp1)	Highly similar to Hcp protein
	VasK-4	NP_931395	1116 aa	4,927,191-4,930,550	18% (*V. cholerae* VasK)	Similar to putative protein of *Y. pestis,* IcmF homologue
	EvpI-5	NP_931403	792 aa	4,938,603-	21.6% (*E.*	Similar to VgrG

				4,940,981	*tarda* EvpI)	related protein
	EvpH-3	NP_931404	893 aa	4,940,978-4,943,659	45.8% (*E. tarda* EvpH)	Similar to ClpA-B type chaperone (ATPase)
	VasF-2	NP_931406	214 aa	4,945,601-4,946,245	20% (*V. cholerae* VasF)	Highly similar to unknown protein
	VasE-4	NP_931407	453 aa	4,946,288-4,947,649	28.6% (*V. cholerae* VasE)	Highly similar to unknown protein
	EvpB-4	NP_931408	510 aa	4,947,666-4,949,198	39.5% (*E. tarda* EvpB)	Similar to unknown protein

Supplementary table 2. Two step secretion systems in *P. luminescens*

**System**	**Components**	**NCBI Accession Number**	**Number of amino acids**	**Gene locus on *P. luminescens* chromosome**	**% Identity**	**Function/Structure**
Type II	GspG	NP_929013	190 aa	2,068,346-2,068,918	20% (*V. cholerae* EpsG)	Hypothetical protein similar to type IV prepilin
	GspJ	NP_929013	190 aa	2,068,346-2,068,918	17.9% (*V. cholerae* EpsJ)	Hypothetical protein similar to type IV prepilin
	GspO	NP_929014	264 aa	2,069,076-2,069,870	44% (*Y. enterocolitica* Yts1O)	Type IV prepilin-like protein leader peptide processing enzyme
	GspJ	NP_929015	391 aa	2,069,910-2,071,085	8.3% (*E. coli* GspJ)	Hypothetical protein with some similarities to PilV
	GspH	NP_929015	391 aa	2,069,910-2,071,085	12.9% (*Y. enterocolitica* Yts1H)	Hypothetical protein with some similarities to PilV
	GspD	NP_929019	534 aa	2,072,176-2,073,780	18% (*K. pneumoniae* PulD)	Hypothetical protein similar to PilN, BfpB
	GspE	NP_929022	520 aa	2,075,627-2,077,189	28% (*E. coli* GspE/ *A. hydrophila* ExeE)	Hypothetical protein similar to PilQ, BfpD
	GspF	NP_929023	365 aa	2,077,196-2,078,293	19.1% (*X. campestris* XpsF)	Hypothetical protein similar to BfpE, PilR and toxin coregulated pilus biosynthesis protein E
	Gsp I	NP_930846	140 aa	4,286,072-4,286,494	14.8% (*A. hydrophila* ExeI)	Putative major pilin subunit
	GspJ	NP_930846	140 aa	4,286,072-4,286,494	19.7% (*Y. enterocolitica* Yts1J)	Putative major pilin subunit
	GspH	NP_930846	140 aa	4,286,072-4,286,494	10% (*V. cholerae* EpsH)	Putative major pilin subunit
	GspG	NP_930846	140 aa	4,286,072-4,286,494	22.6% (*E. coli* GspG)	Putative major pilin subunit
	GspE	NP_930847	480 aa	4,286,662-	38.2% (*E.*	Hypothetical

				4,288,104	*coli* GspE)	protein
	GspF	NP_930848	399 aa	4,288,107-4,2893,06	26.3% (*E. chrysanthemi* OutF)	Type IV pilin biogenesis protein
	GspE	NP_928383	517 aa	1,232,966-1,234,519	28.1% (*Y. enterocolitica* Yts1E)	Probable nucleotide binding protein similar to PilQ
	GspF	NP_928384	361 aa	1,234,512-1,235,597	19.5% (*E. coli* GspF)	Hypothetical protein highly similar to PilR protein
	GspO	NP_928386	221 aa	1,236,269-1,236,934	14.9% (*X. campestris* XpsO)	Hypothetical protein probable prepilin peptidase similar to prepilin peptidase PilU protein
Tat	TatA	NP_931583	86 aa	5,158,550-5,158,810	64.8% (TatA *Escherichia coli*)	Sec-independent protein translocase protein
	TatB	NP_931582	147 aa	5,158,103-5,158,546	57.6% (TatB *Escherichia coli*)	Sec-independent protein translocase protein
	TatC	NP_931581	260 aa	5,157,299-5,158,081	75,8% (TatC *Escherichia coli*)	Sec-independent protein translocase protein, TatABCE protein translocation system subunit
	TatD	NP_930060	261 aa	3,356,852-3,357,637	29.4% (TatD *Escherichia coli*)	Hihgly similar to putative deoxyribonuclease YcfH and to probable metal-dependent hydrolase
		NP_927869	257 aa	572,451-573,224	25.6% (TatD *Escherichia coli*)	Tat-like protein, similar to unknown protein YjjV of *Escherichia coli*
	TatE	NP_931583	86 aa	5,158,550-5,158,810	64.8% (TatE *Escherichia coli*)	Sec-independent protein translocase protein
	SecA	NP_930853	903 aa	4,291,546-4,291,257	89% (*E. coli* SecA)	Preprotein translocase subunit SecA, ATPase
	SecB	NP_931393	158 aa	5,612,392-5,612,868	85.6% (*E. coli* SecB)	Preprotein translocase subunit SecB, molecular chaperone
	SecE	NP_927786	127 aa	460,617-461,000	76.4% (*E. coli* SecE)	Preprotein translocase subunit SecE, channel for translocation across IM
	SecY	NP_931868	443 aa	5,488,023-5,489,354	98% (*E. coli* SecY)	Preprotein translocase subunit SecY, channel pore formation with SecE and

Sec translocase						SecG
	SecD	NP_931103	615 aa	4,573,541-4,575,388	82% (*E. coli* SecD)	Preprotein translocase subunit SecD, complex formation with SecF and YajC that stimulates the pmf protein translocation
	SecF	NP_931102	322 aa	4,572,562-4,573,530	77.4% (*E. coli* SecF)	Preprotein translocase subunit SecF, complex formation with SecD and YajC that stimulates the pmf protein translocation
	SecG	NP_931696	112 aa	5,294,034-5,294,372	76.8% (*E. coli* SecG)	Preprotein translocase subunit SecG
	YajC	NP_931104	109 aa	4,575,416-4,575,745	83.6% (*E. coli* YajC)	Preprotein translocase subunit YajC, links the SecD/SecF/YajC/ YidC complex with the SecY/SecE/SecG Complex
	YidC	NP_932057	546 aa	5,686,149-5,687,789	74.7% (*E. coli* YidC)	Putative IM protein translocase component YidC
SPases	LepB	NP_930561	326 aa	3,968,282-3,969,262	58.7% (*E. coli* LepB)	Signal peptidase I, cleavage of the N-terminal signal peptide of the secreted protein
	LspA	NP_927940	167 aa	674,775-675,278	68.9% (*E.coli* LspA)	Lipoprotein signal peptidase
	BfpA	NP_929014	264 aa	2,069,076-2,069,870	26.6% (*E.coli* BfpA)	Type IV prepilin-like proteins signal peptide processing enzyme
SRP	Ffh	NP_928567	453 aa	1,454,569-1,455,930	87.6% (*E. coli* Ffh)	Signal recognition particle protein
	FtsY	NP_931296	424 aa	4,792,359-4,793,633	65% (*E.coli* FtsY)	Cell division protein, SRP GTPase
Type V (AT)	EstA	NP_930727	649 aa	4,101,200-4,103,149	23% (*P. aeruginosa* EstA)	Lipase 1 precursor (triacylglycerol lipase)
Two-partner system	TpsA1	NP_927589	1,719 aa	230,329-235,488	20% (*S. marcescens* Sh1A)	Some similarities with hemagglutinin/ hemolysin-related protein. Putative transmembrane protein
	TpsB1	NP_927590	559 aa	235,518-237,197	27.8% (*S. marcescens* Sh1B)	Similar to hemolysin activation/ secretion protein
	TpsA2	NP_928461	2,937 aa	1,327,789-	17.3% (*S.*	Some similarities

				1,336,602	*marcescens* Sh1A)	with hemagglutinin/ hemolysin-related protein. Putative transmembrane secreted protein
	TpsB2	NP_928462	554 aa	1,336,651-1,338,315	24.5% (*S. marcescens* Sh1B)	Similar to hemolysin secretion/ activation protein
	TpsA3	NP_927675	1,480 aa	334,184-338,626	42.5% (*S. marcescens* Sh1A)	Hemolysin PhlA
	TpsB3	NP_927676	555 aa	338,692-340,359	60.8% (*S. marcescens* Sh1A)	PhlA hemolysin secretion/ activation protein PhlB
	TpsA4	NP_927898	4,582 aa	604,770-618,518	20.1% (*B. pertussis* FhaA)	Probable hemolysin/ adhesion, similar to hemagglutinin/ hemolysin-related protein
	TpsB4	NP_927899	563 aa	618,577-620,268	22.8% (*B. pertussis* FhaB)	Probable hemolysin secretion/ activation Protein
	TpsA5	NP_928667	2,961 aa	1,629,806-1,638,691	20.9% (*B. pertussis* FhaA)	Probable hemagglutinin secreted protein, similar to hemagglutinin-like secreted protein
	TpsB5	NP_928668	554 aa	1,638,740-1,640,404	22.8% (*B pertussis* FhaB)	Similar to hemolysin secretion/ activation Protein
	TpsA6	NP_930925	3,027 aa	4,374,057-4,383,140	21.4% (*B pertussis* FhaA)	Similar to hemagglutinin/ hemolysin-related proteins, putative transmembrane protein
	TpsB6	NP_930926	554 aa	4,383,189-4,384,853	22.5% (*B pertussis* FhaB)	Similar to outer membrane hemolysin activator Protein
Chaperone/	Chaperone1	NP_927768	252 aa	442,724-443,482	48.8% (*E. coli* PapD)	Putative fimbrial chaperone
	Usher1	NP_927769	862 aa	443,812-446,400	45.5% (*E. coli* PapC)	Similar to outer membrane usher protein precursor
	Chaperone2	NP_928117	249 aa	892,422-893,171	50.6% (*E. coli* PapD)	MrfD protein
	Usher2	NP_928116	851 aa	889,743-892,298	45.2% (*E. coli* PapC)	Outer membrane usher protein MrfC
	Chaperone3	NP_928125	239 aa	898,142-898,861	28.5% (*E. coli* PapD)	Putative fimbrial chaperone

Usher	Usher3	NP_928129	880 aa	901,098-903,740	28.3% (*E. coli* PapC)	Similar to outer membrane usher protein precursor
	Chaperone4	NP_928134	249 aa	907,349-908,098	27.1% (*E. coli* PapD)	Similar to putative fimbrial chaperone
	Usher4	NP_928136	880 aa	909,069-911,711	28.5% (*E. coli* PapC)	Similar to outer membrane usher protein precursor
	Chaperone5	NP_928330	222 aa	1,180,887-1,181,555	26.8% (*E. coli* PapD)	Similar to putative fimbrial chaperone
	Usher5	NP_928329	837 aa	1,178,368-1,180,881	28.5% (*E. coli* PapC)	Similar to outer membrane usher protein precursor
	Chaperone6	NP_927859	232 aa	556,388-557,086	27.5% (*E. coli* PapD)	Similar to putative chaperone
	Usher6	NP_927858	824 aa	553,888-556,362	26.6% (*E. coli* PapC)	Similar to outer membrane usher protein precursor
	Chaperone7	NP_929418	224 aa	2,542,960-2,543,634	29.8% (*E. coli* PapD)	Putative fimbrial chaperone, similar to hypothetical fimbrial chaperone YraI precursor of *Escherichia coli*
	Usher7	NP_929417	846 aa	2,540,382-2,542,922	26.5% (*E. coli* PapC)	Similar to hypothetical outer membrane usher protein YraJ precursor of *Escherichia coli*
	Chaperone8	NP_927629	239 aa	281,565-282,284	26.1% (*E. coli* PapD)	Similar to putative fimbrial chaperone
	Usher8	NP_927631	876 aa	283,299-285,929	29% (*E. coli* PapC)	Similar to outer membrane usher protein precursor
